# Creutzfeldt-Jakob and Vascular Brain Diseases: Their Overlap and Relationships

**DOI:** 10.3389/fneur.2021.613991

**Published:** 2021-02-25

**Authors:** Yacov Balash, Amos D. Korczyn, Nadejda Khmelev, Anda Eilam, Meital Adi, Ronit Gilad

**Affiliations:** ^1^Department of Neurology, Kaplan Medical Center, Rehovot, Israel; ^2^Department of Neurology, Tel-Aviv University, Tel Aviv, Israel

**Keywords:** Creutzfeldt-Jakob disease, stroke, cerebro-vascular accident, diagnosis, differential diagnosis

## Abstract

Only a few case reports of stroke-like onset of Creutzfeldt-Jakob disease (CJD) have previously been published. We aimed to analyze the neurological, imaging, electroencephalographic (EEG), and laboratory features of patients with this very rare phenomenon. Here, we review the clinical characteristics, onset features, and clinical course variants of stroke-like CJD in 23 such patients. The median age of the patients was 71 years (range: 56–84 years); 12 were women. In 20 patients, CJD was sporadic. Thirteen patients developed apoplexy-like onset of symptoms, whereas the others had prodromal non-specific complaints. Most often the patients manifested with pyramidal signs (*n* = 13), ataxia (*n* = 9), and aphasia (*n* = 8). On MRI DWI sequence, all subjects had abnormal hyperintensities in various parts of the cerebral cortex, striatum, or thalamus, while EEG detected periodic triphasic waves only in 11. CSF 14-3-3 protein and total τ-protein were abnormal in 17 of 23 cases. All patients died, median lifespan being 2 months (range: 19 days−14 months). In conclusion, a complex of clinical, radiological, and laboratory manifestations of stroke-like onset of CJD is outlined. The clinical relationships between CJD and stroke are considered, in an attempt to highlight this rare presentation of an uncommon disease.

## Introduction

Creutzfeldt-Jakob disease (CJD) is a rare neurodegenerative disease caused by accumulated misfolded proteins (prions, PrP^Sc^), with their deposition in the cortex, striatum, and thalamus (1). The clinical hallmarks of the disease are progressive dementia, behavioral disorders, epileptic seizures, ataxia, myoclonus, and a variety of other movement disorders (2). Stroke-like onset of CJD is considered as very rare. Indeed, only a single case has been identified in a recent series of cases from China (3). But in an earlier important review 5.6% (30/532) of patients with “probable” or “definite” CJD collected in the UK from 1970 to 1993 had stroke-like onset (4).

In recent years, new observations of this neuropsychiatric syndrome have been reported using improved imaging, laboratory, and genetic methods. However, they were presented as scattered case reports.

To summarize and supplement the clinical picture of stroke-like appearance of CJD and to identify its relationships with actual cases of cerebrovascular accidents, we analyzed all published cases of the disease available in the literature and added a case seen by us.

## Methods

### Patients and Procedures

The clinical information was obtained from the published English and Japanese literature over the past 25 years and is supplemented by one original case. Cases were identified in EMBASE, PubMed, and Scopus biomedical databases, using keywords: Creutzfeldt-Jakob disease AND stroke, cerebro-vascular accident.

Inclusion criteria were as follows: (1) clinical situation when the onset of CJD was initially misdiagnosed as a stroke; (2) subsequent workup indicated probable or definite CJD according to the international criteria (5); or (3) detailed clinical case description including MRI, electroencephalogram (EEG), and cerebrospinal fluid (CSF) studies when available were consistent with the diagnosis of CJD. Data on brain biopsy and/or autopsy data, if performed, were included.

### Statistical Analysis

Data were analyzed using a Microsoft Excel 2010 spreadsheet. Results were expressed as means with standard deviations (SD) or as median with interquartile range (IQR).

## Results

Twenty-three clinical cases (cases 1–23) were analyzed. The mean age of the subjects was 70.1 years (SD = 7.3, median = 71, IQR: 66–75), of whom 12 (52.2%) were females. In 20 cases, sporadic CJD was diagnosed, in two cases, the disease was familial [cases 6 (6) and 7 (7)], and in another case, a mutation was identified characteristic of familial CJD, but no other cases were reported in his family [case 20 (8)] (Table 1).

**Table 1:** Demographic characteristics and clinical features in 23 patients with stroke-like onset of CJD.

Females	12
Median age, range (years)	71, 56–84
Sporadic CJD	19
Familial CJD	2
Genetic, no family history	1
Beginning with apoplectic-like or wake up symptoms (number and %)	13 (56.5%)
Beginning with prodromal symptoms (number and %)	10 (43.5%)

### Clinical Vignette (Case 23)

**Figure 1:**
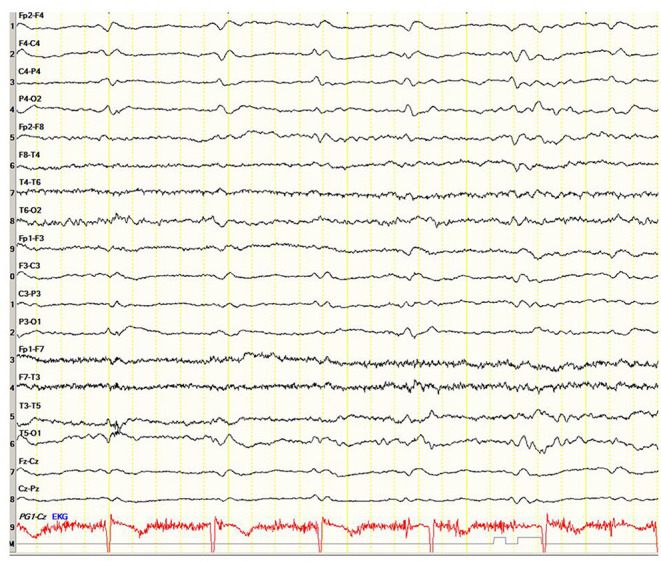
Case 23. Electroencephalogram showing periodic triphasic sharp wave complexes mostly at the right fronto-parietal area.

**Figure 2:**
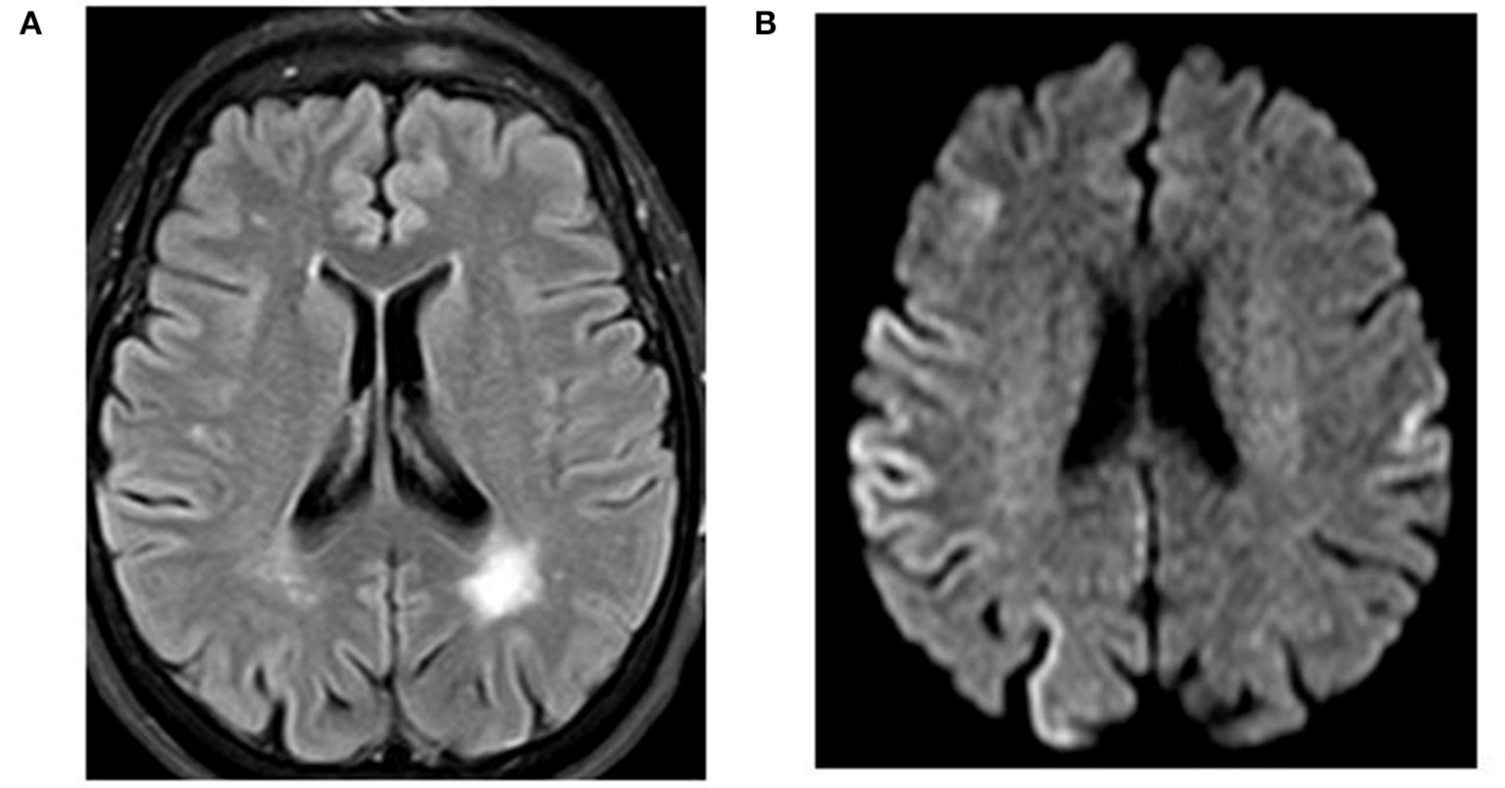
Case 23. Magnetic resonance imaging of the brain. **(A)** FLAIR indicating a left parietal lobe old infarction. **(B)** Diffusion-weighted magnetic resonance imaging with restricted diffusion at the right parietal cortex.

A 71-year-old right-handed woman was evaluated in the emergency department with a 2-day history of asthenia, dizziness, gait instability, speech disorders, and periodic involuntary jerky movements in her left arm. Past medical history included mild well-controlled hypertension. On admission, she was disorientated, her cranial nerves were normal, she had mild weakness and jerky dystonia with hyperactive tendon reflexes, and suspected hemi-ataxia were seen in the left arm. Plantar responses were flexor. A non-enhanced CT scan revealed a left parietal hypodense focus and cerebellar calcifications, which could not explain ipsilateral symptoms. At this stage the patient was clinically diagnosed with possible ischemic stroke in the right hemisphere. However, over a 3-week hospitalization period, her condition progressively deteriorated. She became negativistic and non-compliant. A psychiatric examination revealed inappropriate affect with euphoria and visual hallucinations. Her MoCA (9) score (day 5) was 19/30 and later declined to 16/30 (day 12). The myoclonic jerks (Supplementary Video 1) in the left arm were accompanied by rigidity, general weakness, incontinence, and eventually inability to stand and walk. No visual impairment or startle responses were seen. Repeated EEG (days 2, 5, and 10) disclosed diffuse delta activity with periodic triphasic sharp wave complexes mostly at the right fronto-parietal area in later records (days 5 and 10) (Figure 1). A brain MRI (day 6) showed multiple bilateral microvascular and macrovascular ischemic periventricular white matter lesions. DWI imaging (day 8) revealed restriction in diffusion with ribbon signs in the right parietal cortex (Figures 2A,B). This raised suspicion toward a stroke-like onset CJD.

CSF exhibited no cells; glucose was 72 mg/dl, protein 34 mg/dl. However, total τ-protein was markedly elevated up to 2,524 pg/ml (normal level <240 pg/ml), suggesting a diagnosis of probable sporadic CJD according to the updated diagnostic criteria (5). Other laboratory data showed normal hematologic, biochemical, endocrine, and vitamin levels. Autoimmune workups, viral and bacterial screens including HIV and treponema pallidum, as well as a paraneoplastic antibodies panel, was negative. The patient was discharged with the diagnosis of probable sporadic CJD and died in a *nursing home* nearly 3 weeks later. Autopsy has not been performed.

**Figure 3:**
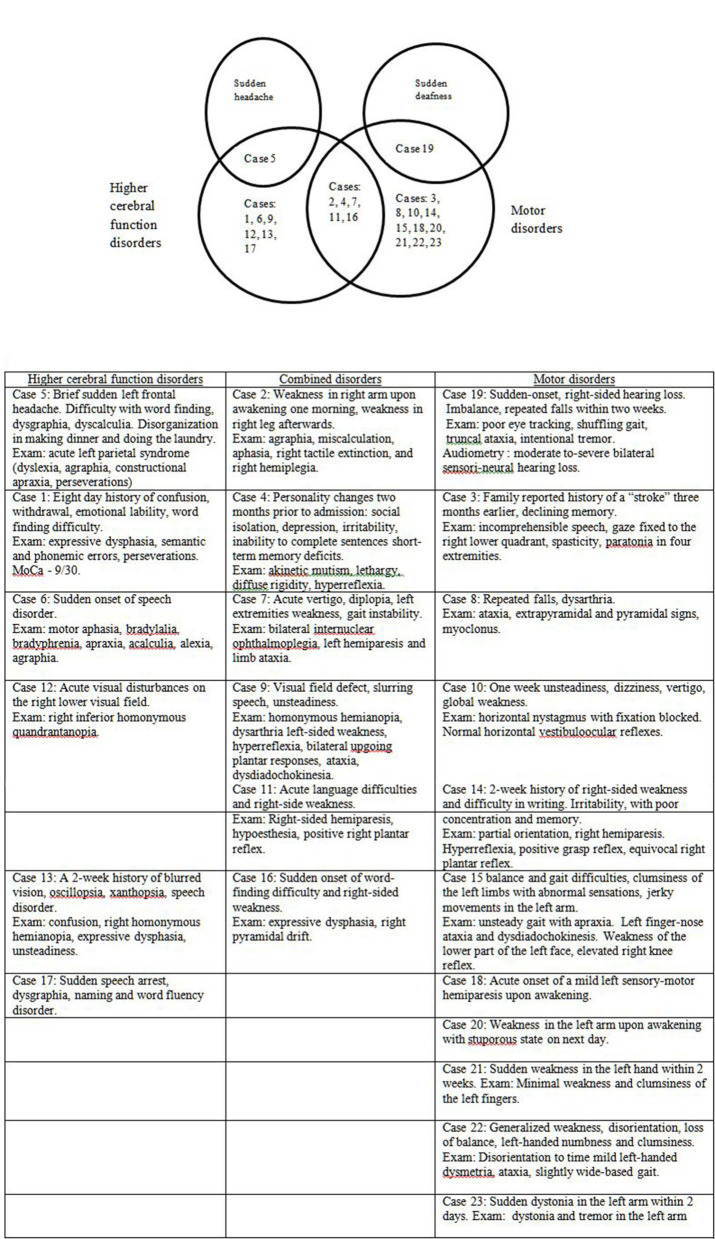
Initial neurological symptoms and signs in 23 patients with stroke-like onset of CJD.

Thirteen patients (56.5%) in this series presented with sudden apoplectic-like or wake up symptoms and (visual field defect or double vision, speech disorder, acute vertigo, and sudden hearing loss), motor disorders mono- or hemiparesis, dysmetria, dystonia, alien hand, or combined disorders (Figure 3). Ten other patients (43.5%) reported prodromal complaints (weakness, insomnia, personality changes, dysarthria, word finding difficulty, memory impairment or confusion, dizziness, and unsteadiness) lasting from several weeks to 6 months prior to an acute stroke-like deterioration, which in retrospect could be ascribed to incipient onset of CJD. Sixteen patients (69.6%) had one or more vascular risk factors most commonly hypertension (11 individuals, 47.8%), and two of them (8.7%) had a history of previous stroke or recurrent stroke-like symptoms [cases 4 (10), 9 (11)]. In several cases [7 (7), 13 (12), 16 (13), 18 (14), 20 (8)] the patients were discharged for outpatient or rehabilitation treatment after a temporary stabilization of their condition over 2–8 weeks.

Other differential diagnoses were antibody-mediated autoimmune encephalitis [cases 4 (10) and 11 (15)] treated with methylprednisolone pulse therapy and non-convulsive status epilepticus due to sharp delta activity [case 5 (16)] treated with levetiracetam and phenytoin.

The vast majority of patients, 20/23 (87%), showed a rapid progressive deterioration after the acute onset in both motor and cognitive state, followed by unresponsiveness, blindness, akinetic mutism, stupor, ataxia, generalized rigidity, spasticity, hyperreflexia, and myoclonus. In other 3 patients (13%) [cases 7 (7), 14 (17), 20 (8)] the deterioration to bedridden and mute condition was prolonged, lasting 9-14 months.

The mean survival of the 23 patients from the stroke-like onset was 4.2 months (*SD* = 4.1, median = 2, IQR: 1.1–5), range 19 days−14.2 months.

Brain MRI studies were performed in 22/23 patients (95.6%) and in 13 of them (59.1%) within 1–14 days from the onset of CJD symptoms. Four patients in this group had MRI not characteristic for CJD (18.2%). In four other patients, non-enhanced MRI images revealed unilateral or bilateral diffuse areas of abnormal high signal intensities with restriction in diffusion (ribbon sign) in various parts of the cerebral cortex (18.2%), and in the remaining five patients (22.7%), there was a combined lesion of the cortex and basal ganglia (hyperintensity of the head of caudate nucleus and putamen) on DWI sequence. In the remaining nine patients (40.9%), MRI images were performed later within two months from the onset of the apoplectic presentation. All of them were pathological, often with combined lesions of various parts of the cortex and basal ganglia (caudate nucleus, thalamus, and lentiform nucleus) (see Table 2).

**Table 2:** Ancillary tests in 23 patients with stroke-like onset of CJD.

**Brain MRI (DWI) within first 2 weeks (information for 13 patients)**	
Unremarkable for CJD	4
Predominantly cortical hyperintensity	4
Predominantly basal ganglia hyperintensity	0
Combined CJD-related	5
**Brain MRI (DWI) in subsequent period on average after 2 months (information for 9 patients)**	
Unremarkable for CJD	0
Predominantly cortical hyperintensity	3
Predominantly basal ganglia hyperintensity	1
Combined CJD-related	5
**EEG within first 2 weeks (information for 10 patients)**	
Periodic triphasic waves	2
Abnormal non-specific brain activity	6
Normal brain activity	2
**EEG in subsequent period (information for 13 patients)**	
Periodic triphasic waves	6
Abnormal non-specific brain activity	6
Normal brain activity	1
**CSF proteins (information for 19 patients)**	
Protein 14-3-3 positive	11/16
Total τ-protein positive	6/7
RT-QuIC assay positive for PrP^Sc^	3/3
S100b protein positive	3/3
E200K mutation positive	Case 7
V180I mutation positive	Case 20

Along with the above mentioned MRI signs characteristic of CJD, in 4/22 cases (18.2%), T2 FLAIR in the first 3–4 days detected only scattered hyperintensities in the centrum semiovale [cases 8 (18), 9 (11), 15 (19), and 22 (20)] and in two other patients [cases 7 (7) and 19 (21)] DWI disclosed a combination of cortical ribboning hyperintensites with non-specific periventricular vascular changes. In the abovementioned studies [cases 7 (7) and 19 (21)], MRI performed during the first days revealed only an acute lesion at the left pontine-midbrain border compatible with an ischemic stroke and small vessel ischemic changes in periventricular areas of the brain but were otherwise normal. In case 7 (7), repeated MRI studies 3, 5, and 8 months after symptoms onset showed increasing DWI hyperintense lesions in the thalamus, basal ganglia, and anterior cingulate together with the gradual shrinkage and disappearance of the ischemic brainstem lesion in the last MRI (Table 2).

In one case, MRI changes characteristic of CJD were detected one month before the acute onset of the clinical picture, but the diagnosis was not made at that time due to the absence of clinical symptoms other than repeated falls [case 8 (18)].

Single or repeated EEG were recorded in all 23 studies. During the first 2 weeks after the CJD symptoms onset, the initial recordings were performed in 10/23 patients (43.5%). In two subjects, the EEG was within the normal range, in six, non-specific changes were registered (diffuse slowing with theta–delta activity, generalized dysrhythmia, focal abnormalities, and epileptiform discharges), while periodic sharp waves were observed in only two individuals. In a later period, on average 55 ± 4.5 days, in 6/23 patients (26.1%), the EEG changes remained non-specific, whereas in 6/23 cases, periodic triphasic waves were recorded (26.1%). In one patient, the EEG remained normal at 7 months after the onset of the disease shortly before death [case 8 (18)] (Table 2).

CSF 14-3-3 protein was studied in 16 patents and was positive in 11 (68.8%), τ-protein was measured in seven patients and was elevated on average to 1,924 ± 1,459 pg/ml in six cases (range: 789–4,219). RT-QuIC assay was positive in three cases: 1 (22), 2 (23), and 4 (10). S100B CSF protein was positive in three cases: 1 (22), 3 (24), and 9 (11).

Genetic analysis was abnormal in case 7 (7) (E200K). One other case revealed Val–>Ile at codon 180 [case 20 (8)].

Autopsy was performed in 9/23 cases (39.1%) and confirmed spongiform encephalopathy with diffuse synaptic pattern of prion protein immunoreactivity. None of them had changes consistent with an acute stroke. A brain biopsy, which verified the diagnosis, was performed in one patient. In general, these pathologically confirmed cases (Table 3) were not different from the other 13 patients.

**Table 3:** Brain autopsy or biopsy data in 10 patients with apoplectic onset of CJD.

**Case** ** number**	**Age**	**Gender**	**Beginning type**	**Sporadic or** ** Familial**	**Brain autopsy or biopsy data**
3	74	female	prodromal	sporadic	3F4 immunostaining with characteristic features of spongiform encephalopathy.
6	65	male	apoplectic	familial	Cortical spongiosis, neuronal loss, astrogliosis, pathological prion protein detected
11	78	male	apoplectic	sporadic	CJD confirmed
13	75	male	prodromal	sporadic	Spongiform degeneration in the parietal and occipital cortex, with a prion protein deposition
15	67	female	prodromal	sporadic	Spongiform degeneration with vacuolar change in the molecular layer of the cerebellum and, in neocortical areas, putamen, and thalamus. Immunohistochemistry showed protease-resistant prion protein immunoreactivity, most clearly in the cerebellar cortex, in neocortical and basal ganglia specimens
17	62	male	apoplectic	sporadic	Spongiform encephalopathy. Immunoblot: protease-resistant scrapie prion protein (PrPS^c^)
18	75	male	apoplectic	sporadic	Spongiform encephalopathy and a diffuse synaptic pattern of prion protein immunoreactivity
19	74	female	prodromal	sporadic	Brain biopsy: presence of protease resistant prion protein
21	72	male	apoplectic	sporadic	CJD confirmed
22	73	male	prodromal	sporadic	Extensive microvacuolation through the cortex, striatum, and hippocampus as well as diffuse PrP immunostaining

*CJD, Creutzfeldt-Jakob disease; 3F4, Anti-prion protein antibody; PrPS^c^, misfolded version of the prion protein*.

## Discussion and Conclusions

In our case, as in the other cases cited, the sudden onset of neurological disorders suggested an acute stroke, but further developments and tests revealed the diagnosis of CJD.

The available data on the relationship between CJD and stroke, suggest several possibilities.

The apoplectic onset, presumably caused by local neuronal shutdown induced by rapid prion propagation, was initially diagnosed as stroke, and only later progressive clinical worsening supplanted by MRI, EEG, or CSF data indicated prion disease. No evidence for acute vascular brain lesion was observed on initial CT or MRI scans. This situation occurred in the majority of the cited cases: 1 (22), 4 (10), 6 (6), 8 (18), 10 (25), 12 (26), 13 (12), 14 (17), 17 (27), 20 (8), 21 (28), and 22 (20).

In our subject (case 23), as in some others [cases 2 (23), 9 (11), 15 (19), 16 (13), and 18 (14)], stroke-like CJD was initially diagnosed clinically as true vascular disease of the brain and CT or MRI imaging have suggested acute or chronic cerebrovascular disease. However, further clinical deterioration which occurred within 3–4 weeks forced the revision or repeat of MRI, which now revealed DWI cortical or subcortical hyperintensities and/or elevated CSF τ-protein or positive 14-3-3 protein. On initial MRI in some patients [cases 5 (16), 7 (7), 9 (11), and 19 (21)] with stroke-like onset CJD, there could be visible only vascular changes on FLAIR MRI studies without DWI features characteristic of CJD.

One patient was diagnosed with a stroke with progressive neurological deterioration. After her death, however, this diagnosis was not confirmed at autopsy, and instead, showed positive 3F4 immunostaining with characteristic features of spongiform encephalopathy along with *PRNP* gene sequence analysis with 129 polymorphism valine homozygosity (VV2) [case 3 (24)].

In another case, the clinical picture was apoplectic-like, the MRI data indicated CJD, showing abnormal high signal intensities in the contralateral frontal cerebral cortex, but both CSF protein 14-3-3 and *PRNP* gene analysis were negative. The diagnosis of CJD was established post-mortem with *PRNP* gene analysis where subtype MM/MV1 + 2C was uncovered [case 11 (15)].

Thus, the diagnosis of CJD should be considered in every stroke-like patient in whom the suspicion of stroke is not confirmed by MRI, particularly if a history of recent progressive cognitive, behavioral, or movement disorders can be elicited. Of course, the characteristic changes in DWI and FLAIR MRI should alert the clinicians to this possibility. In such situations, CSF τ-protein or RT-QuIC assay should be examined and repeat MRI should be performed.

It is remarkable that all the cases described in the literature had a stroke-like incidence of CJD at the disease onset. We have not seen a description of a stroke-like deterioration in a patient already diagnosed as CJD. However, this probably reflects reporting bias.

The causes of stroke-like onset CJD remain unknown. Possibly, they are related to the rate of prions accumulation, for which there may be a genetic reason (15). However, the *PRNP* gene sequence analysis in patients with stroke-like sporadic CJD was performed only in 2/23 studies [case 3 (24)—VV2 at the polymorphic codon 129; case 11 (15)—subtype MM/MV1 + 2C PrP^Sc^-type accumulation, (29)], which did not allow us to draw any conclusions.

Currently, the gold standard method for definitive CJD diagnosis during life remains a brain biopsy. This is rarely done due to the risk of additional brain damage in the absence of curative treatment of the disease. Therefore, the developments of less invasive methods of intravital tissue diagnostics for prion diseases, such as olfactory mucosa washout or skin biopsies, remain relevant (30).

In general, our conclusions are similar to those of McNaughton and Will (4) from over 20 years ago, but the cases reported here show a wide spectrum of MRI results.

As expected in a retrospective case series, not all patients have been investigated in the same way. Moreover, this series cannot be regarded as representative. We presume that many cases are not diagnosed correctly and others are not reported.

This series of cases is reported in order to increase awareness of the possibility that CJD may present in an apoplectic form, which may lead to erroneous management. Since MRI is now more commonly performed in acute stroke, the characteristic ribbon-like cortical signal should indicate the correct diagnosis. While we have focused on the acute onset of symptoms, probably an important red flag should be the progressive clinical deterioration of the patient afterwards.

In conclusion, CJD may have an apoplectic onset and clinicians should be alert to this possibility to avoid the consequences of misdiagnosis.

## Consent for Publication

We obtained written informed consent for publication from the family of our patient (case 23).

## Author Contributions

YB contributed to the conception, execution, design, and preparation of the manuscript for publication. AK contributed to the conception, design, and review of the manuscript. NK contributed to the execution and preparation of the manuscript for publication. AE contributed to the execution and review of the manuscript. RG contributed to the conception, execution, and review of the manuscript. MA contributed to the design and review of the manuscript. All authors contributed to the article and approved the submitted version.

## Conflict of Interest

The authors declare that the research was conducted in the absence of any commercial or financial relationships that could be construed as a potential conflict of interest.

## Supplementary Material

Supplementary Video 1 Case 23. Myoclonic jerks in the left shoulder and arm in 71 year old woman with probable CJD.

## Abbreviations

CJD: Creutzfeldt-Jakob's disease
CSF: cerebrospinal fluid
CT: computed tomography
CVA: cerebrovascular accident
DWI: diffusion-weighted magnetic resonance imaging
EEG: electroencephalogram
FLAIR: fluid-attenuated inversion recovery imaging
HIV: the human immunodeficiency viruses
MoCA: Montreal Cognitive Assessment
MRI: magnetic resonance imaging
PRNP: (prion protein) gene encoding for the major prion protein
PrP^Sc^: scrapie isoform of the prion protein
RT-QuIC: real-time quaking-induced conversion assay.
